# The energy metabolism-promoting effect of aconite is associated with gut microbiota and bile acid receptor TGR5-UCP1 signaling

**DOI:** 10.3389/fphar.2024.1392385

**Published:** 2024-09-11

**Authors:** Dandan Zhang, Hao Cheng, Jing Wu, Yaochuan Zhou, Fei Tang, Juan Liu, Wuwen Feng, Cheng Peng

**Affiliations:** ^1^ State Key Laboratory of Southwestern Chinese Medicine Resources, School of Pharmacy, Chengdu University of Traditional Chinese Medicine, Chengdu, China; ^2^ TCM Regulating Metabolic Diseases Key Laboratory of Sichuan Province, Hospital of Chengdu University of Traditional Chinese Medicine, Chengdu, China; ^3^ Key Laboratory of the Ministry of Education for Standardization of Chinese Medicine, Chengdu University of Traditional Chinese Medicine, Chengdu, China; ^4^ School of Basic Medical Sciences, Chengdu University of Traditional Chinese Medicine, Chengdu, China

**Keywords:** aconite, gut microbiota, bile acids, antibiotic, fecal microbiota transplantation, energy metabolism

## Abstract

**Introduction:**

As a widely used traditional Chinese medicine with hot property, aconite can significantly promote energy metabolism. However, it is unclear whether the gut microbiota and bile acids contribute to the energy metabolism-promoting properties of aconite. The aim of this experiment was to verify whether the energy metabolism-promoting effect of aconite aqueous extract (AA) is related to gut microbiota and bile acid (BA) metabolism.

**Methods:**

The effect of AA on energy metabolism in rats was detected based on body weight, body temperature, and adipose tissue by HE staining and immunohistochemistry. In addition, 16S rRNA high-throughput sequencing and targeted metabolomics were used to detect changes in gut microbiota and BA concentrations, respectively. Antibiotic treatment and fecal microbiota transplantation (FMT) were also performed to demonstrate the importance of gut microbiota.

**Results:**

Rats given AA experienced an increase in body temperature, a decrease in body weight, and an increase in BAT (brown adipose tissue) activity and browning of WAT (white adipose tissue). Sequencing analysis and targeted metabolomics indicated that AA modulated gut microbiota and BA metabolism. The energy metabolism promotion of AA was found to be mediated by gut microbiota, as demonstrated through antibiotic treatment and FMT. Moreover, the energy metabolism-promoting effect of aconite is associated with the bile acid receptor TGR5 (Takeda G-protein-coupled receptor 5)-UCP1 (uncoupling protein 1) signaling pathway.

**Conclusion:**

The energy metabolism-promoting effect of aconite is associated with gut microbiota and bile acid receptor TGR5-UCP1 signaling.

## 1 Introduction

Energy metabolism is a basic and important way for the body to obtain and utilize energy to maintain normal functions. Adipose tissue, a tissue with high energy metabolic activity, is crucial for regulating the balance of energy metabolism in the whole body. Adipose tissues can be divided into white adipose tissue (WAT) and brown adipose tissue (BAT) according to different functions (Cohen and Kajimura, 2021). WAT is responsible for storing energy in the form of fat (Machado et al., 2022). Different from WAT, BAT is a thermogenic tissue that mainly relies on its specific UCP1 (uncoupling protein 1) thermogenesis to upregulate energy expenditure, which is central to energy metabolism (Choe et al., 2016). Interestingly, WAT also has the ability to promote energy metabolism, similar to BAT, through a process called browning of WAT. Due to its high plasticity, adipose tissue can reshape itself via changes in the number and/or size of adipocytes in response to changes in the body’s energy state. Hence, enhancing the thermogenic activity of BAT and promoting the browning of WAT are strategies to improve energy metabolism.

Gut microbiota has gained much attention in recent years, and accumulating research studies have found that gut microbiota and its metabolites can regulate host energy metabolism (Heiss and Olofsson, 2018; Molinaro et al., 2018; Fan and Pedersen, 2021). Bile acids (BAs) are a class of metabolites that are produced from cholesterol in the liver and then modified by the gut microbiota. Primary BAs are synthesized mainly by the classical and alternative pathways in the liver (Molinaro et al., 2018). After being discharged into the gut, they are converted into secondary BAs by gut microbiota through decoupling, dehydrogenation, dihydroxylation, and isomerization (Molinaro et al., 2018). By binding to the corresponding receptors, BAs can mediate signaling pathways in response to energy metabolism. Takeda G-protein-coupled receptor 5 (TGR5) is a membrane surface receptor expressed in BAT and WAT. BAs can activate the cAMP (the cyclic adenosine monophosphate)/PKA (protein kinase A) signaling pathway via the stimulation of TGR5, thereby upregulating UCP1 expression in BAT and WAT and promoting energy metabolism (Chiang, 2013; Cai et al., 2022).

Aconite, the processed product of the lateral root of *Aconitum carmichaelii* Debx., is a traditional Chinese medicine (TCM) with hot properties (Singhuber et al., 2009; Zhou et al., 2015). According to the theory of TCM, aconite can promote energy metabolism (Singhuber et al., 2009). After oral administration, the active components of TCM, especially those with low bioavailability, are difficult to be absorbed and interact with gut microbiota to exert their efficacy (Feng et al., 2019). Prior research studies have demonstrated that aconite can interact with the gut microbiota upon entering the gut, and thus, the pharmacological effects of aconite can be affected (Zhen et al., 2021; Liu et al., 2023; Tu et al., 2023; Zhang et al., 2023). Therefore, the gut microbiota may account for promoted energy metabolism induced by aconite. Considering the effect of gut microbiota and BAs on BAT activation and WAT browning, modulating gut microbiota and BAs may be an important strategy to promote energy metabolism in adipose tissue. Therefore, we attempted to investigate the mechanism by which AA promotes energy metabolism and elucidate the potential role of the gut microbiota–BA–TGR5-UCP1 axis in mediating these therapeutic effects of AA.

## 2 Material and methods

### 2.1 Preparation of the material

Aconite was prepared and analyzed in accordance with the methods described above (Liu et al., 2021). Aconite was purchased from Sichuan Jiangyou Zhongba AA Technology Development Co., Ltd. (Jiangyou, China). The weighed aconite was soaked for 30 min in water at 10-fold dilution before extraction. Aconite was boiled for 5 h and then filtered, added to eight times of water to the filter residue, cooked for 3 h, and then filtered. The two filtrates were combined and concentrated at 60°C under reduced pressure. Appropriate rosiglitazone tablets were taken and dissolved in pure water for later use. Ultra-high-performance liquid chromatography coupled with quadrupole time-of-flight mass spectrometry (UPLC–QTOF/MS) and high-performance liquid chromatography (HPLC) were used to study the chemical compounds of AA. Their specific compounds and contents can be found in our previous study (Liu et al., 2021).

### 2.2 Animals and experimental protocol

Specific-pathogen-free (SPF)-grade adult male SD rats (180–200 g) were purchased from SiPeiFu (Beijing) Biotechnology Co., Ltd. (SCXK (jing) 2019–0010). The rats were housed under a standard environment (22°C ± 2°C, 12-h light/12-h dark cycles, and free access to water and food). All experiments were approved by the Animal Ethics Committee of Chengdu University of Traditional Chinese Medicine (approval no. SYXK (chuan) 2020-124).

#### 2.2.1 Animal experiment 1

A total of 24 rats were adaptively housed for 7 days and randomly divided into three groups (n = 8 per group): Ctrl (control) group (distilled water), AA group (6.2 g/kg), and RSG (rosiglitazone) group (RSG 2 mg/kg). The experiment lasted for 21 days, and the rats were weighed every 3 days.

#### 2.2.2 Animal experiment 2: antibiotic (ABX) experiments

A total of 16 rats were adaptively housed for 7 days and randomly distributed into two groups (n = 8 per group): Ctrl-ABX group and AA-ABX group. The Ctrl-ABX group was given an antibiotics cocktail (ABX) (0.5 g/L vancomycin, 1 g/L metronidazole, 1 g/L ampicillin, and 1 g/L neomycin sulfate) along with a normal diet for 21 days. The AA-ABX group was given ABX along with AA (6.2 g/kg) for 21 days. The rats were weighed every 3 days.

#### 2.2.3 Animal experiment 3: fecal microbiota transplantation (FMT)

A total of 32 rats were adaptively housed for 7 days and randomly distributed into four groups (n = 8 per group): donor–Ctrl group, donor–AA group, recipient group receiving FMT from the Ctrl group (Ctrl–FMT), and recipient group receiving FMT from the AA group (AA–FMT). Rats in the recipient groups were treated with ABX for 7 days to eliminate gut microbiota before administration of the corresponding solution. The rats in the Ctrl group were fed with distilled water, and rats in the AA group were fed with AA (6.2 g/kg); the whole process lasted for 14 days. During this period, rat feces were collected every 2 days for the FMT experiment. Specifically, rats in the recipient Ctrl-FMT group received feces from donor-Ctrl rats, and rats in the recipient AA-FMT group received feces from donor-AA rats. The FMT experiment was conducted based on a previous study, with partial modifications (Zhou et al., 2021). Specifically, fresh feces from each group of donor rats were gathered and vigorously mixed in sterile saline (fecal/sterile saline = 1:5, w/v) using a benchtop vortex. The mixture was centrifuged at 800 *g* for 10 min, and the supernatant was collected for transplantation. Recipient rats were given 2 mL fresh transplant supernatant by oral gavage daily for 14 days. The body weight was measured every 2 days. Note that fresh transplanted fecal materials were prepared within 10 min prior to oral gavage administration at the time of transplantation to prevent changes in microbial composition.

### 2.3 Acute exposure to cold

After the end of the administration time, all rats in the three separate experiments were subjected to cold exposure. The rats were placed in a fridge (4°C) for 4 h with free access to food and water.

### 2.4 Measurement of temperature

Rectal temperature (the core body temperature) was measured using a thermometer before and after cold exposure, and the length of the thermometer inserted into the rectum was approximately 1.5 cm. The body temperature of the rats on other surfaces (eyes, dorsal, and ventral) was recorded by infrared cameras (FLIR ONE ANDROID, United Kingdom) and analyzed with FLIR Tools.

### 2.5 Weight, histopathology, and immunohistochemistry of adipose tissue

BAT was obtained from the scapula, and WAT was obtained from the groin; they were then cleaned with normal saline, drained with filter paper, and weighed. For histopathology, the collected adipose tissue was fixed with 4% paraformaldehyde solution, then dehydrated and embedded in paraffin, and stained with hematoxylin and eosin (HE). The morphological changes in the adipose tissue were observed by a digital section scanner. For immunohistochemistry (IHC) detection of UCP1, adipose tissue was fixed with 4% paraformaldehyde and then dehydrated, embedded in paraffin, and stained according to the IHC procedure. ImageJ software was used to analyze the adipocyte size in WAT and the expression of UCP1 in WAT and BAT.

### 2.6 Protein extraction and Western blotting analysis

Total protein was extracted from adipose tissue with a protein extraction reagent and then determined with a BCA protein assay kit (both are from Shanghai Epizyme Biomedical Technology Co., Ltd.). The extracted protein samples were separated using SDS-PAGE gels and then transferred to PVDF membranes by the wet-transfer method. After sealing with 5% bovine serum albumin for 2 h at room temperature, the membranes were incubated with the primary antibody at 4°C overnight (anti-TGR5, 1:1,000, ABclonal; anti-PKA, 1:20,000, Abcam; anti- UCP1, 1:1,000, ABclonal; *β*-Tubulin, 1:5,000, ABclonal). The membranes were washed thrice with TBST and incubated at room temperature with the secondary antibody for 2 h. The protein bands on the membrane were observed with enhanced chemiluminescence reagent (Oriscience, China), and the protein bands were visualized and quantified in ImageJ software.

### 2.7 16S rRNA gene sequencing

The fresh feces of the rats were collected in a 1.5-mL centrifuge tube and stored in a −80°C freezer. Total genomic DNA from fecal samples was extracted using the E.Z.N.A.^®^ Soil DNA Kit (Omega Bio-tek, Norcross, GA, United States). The quality of genomic DNA extracted was assessed using 1% agarose gel electrophoresis, and the DNA purity and concentration were determined via NanoDrop2000

The PCR amplification of the 16S rRNA gene V3–V4 variable region was performed using upstream primer 338F (5'ACT​CCT​ACG​GGA​GGC​AGC​AG-3′) and downstream primer 806R (5′-GGACTACHVGGGTWTCTAAT-3′). The NEXTFLEX Rapid DNA-Seq Kit was used to construct purified PCR products. Sequencing and bioinformatics analysis of fecal samples were performed on Illumina’s MiSeq PE300/NovaSeq PE250 platform (Majorbio Bio-Pharm Technology Co. Ltd., Shanghai, China).

### 2.8 Quantification of BAs in feces

Qualitative and quantitative detection of BA in samples is performed by UPLC-MS/MS (UHPLC-Qtrap). A fecal sample of 50 mg was weighed and mixed with 400 μL methanol–water mixture (4:1) for protein precipitation. The sample was centrifuged at 4°C 13,000 g for 15 min, and the supernatant was dried with nitrogen. The samples were separated by BEH C18 (150*2.1 mm, 1.7 μm) liquid chromatography column and then detected by mass spectrometry. The mobile phase was 0.1% formic acid–water solution and 0.1% formic acid–acetonitrile solution. The gradient conditions were as follows: 0–10 min (25%–32% B), 10–26 min (32%–75% B), 26–26 min (75%–100% B), 26–28.0 min (100% B), 28–28.1 min (100%–5% B), and 28.1–32 min (25% B). AB SCIEX quantitative software OS was used for analyzing the raw data.

### 2.9 Statistical analysis

Statistical analysis was carried out in GraphPad Prism software (version 8.0). All data are presented as the mean ± SD. Statistical differences between the three groups were determined by one-way ANOVA (when data were normally distributed) and Kruskal–Wallis tests (when data were not normally distributed). The differences between the two groups were examined using unpaired Student’s t-test. *P*-values <0.05 were considered statistically considerable.

## 3 Results

### 3.1 Effect of AA on body weight and body temperature

**FIGURE 1:**
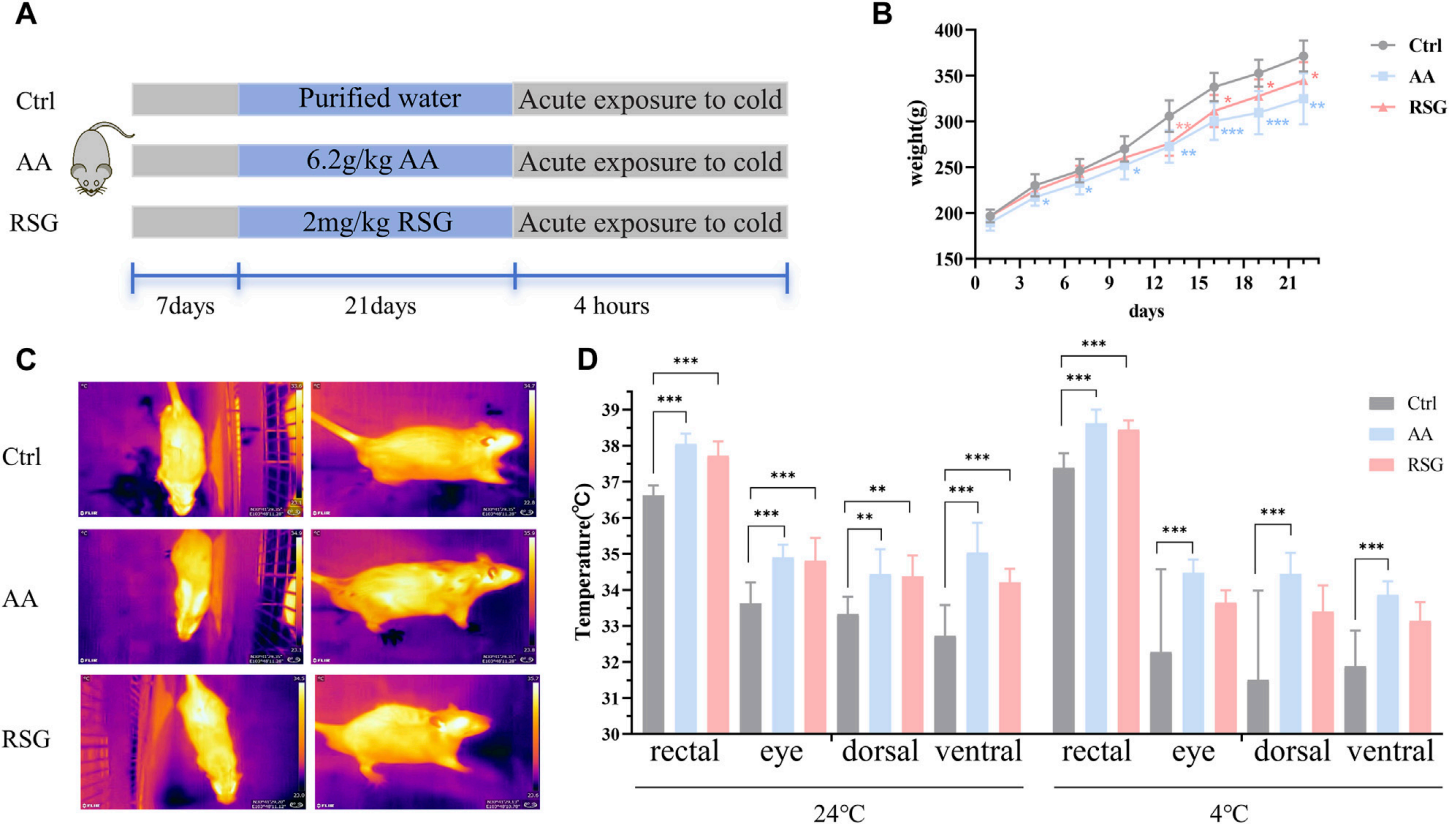
AA reduced body weight gain and enhanced the body temperature. **(A)** Experimental design and timeline. **(B)** Changes in body weight of rats. **(C)** Representative thermal images of rats. **(D)** Body temperature of rats before and after cold exposure. Data are presented as mean ± SD, n = 8. *p < 0.05, **p < 0.01, and ***p < 0.001.

The treatment of each group during the entire experimental process is shown in Figure 1A. After 21 days of administration, the rats in the AA and RSG groups showed a significantly lower weight gain than rats in the Ctrl group (Figure 1B), which preliminary indicated a stimulatory effect of AA on energy metabolism. Since energy metabolism is susceptible to changes in temperature and exposure to cold has been shown to enhance energy metabolism, we conducted an acute cold-exposure tolerance test to evaluate differences in energy metabolism among the three groups of rats. Therefore, rectal temperature (the core temperature) and temperature of different body surface sites were measured at room temperature (24°C) and cold exposure (4°C for 4 h). Results showed that the rectal temperature of the AA and RSG groups remained higher than those in the Ctrl group at 24°C (Figure 1D). The temperature of various body surface locations was measured using infrared thermal imaging, including the eye temperature, the dorsal temperature (which represents the temperature from BAT), and the ventral temperature (which indicates the temperature from WAT). The results showed the same trend as rectal temperature (Figure 1D). After 4 h of cold exposure, the rectal temperature of rats in the AA and RSG groups was still higher than that in the Ctrl group (Figure 1D). The infrared imaging results also showed that the temperature of rats in the AA group was higher than that of rats in the Ctrl group, but there was no significant difference between rats in the RSG and Ctrl groups. These results suggested that AA can promote energy metabolism.

### 3.2 AA promotes thermogenesis of BAT and browning of WAT

**FIGURE 2:**
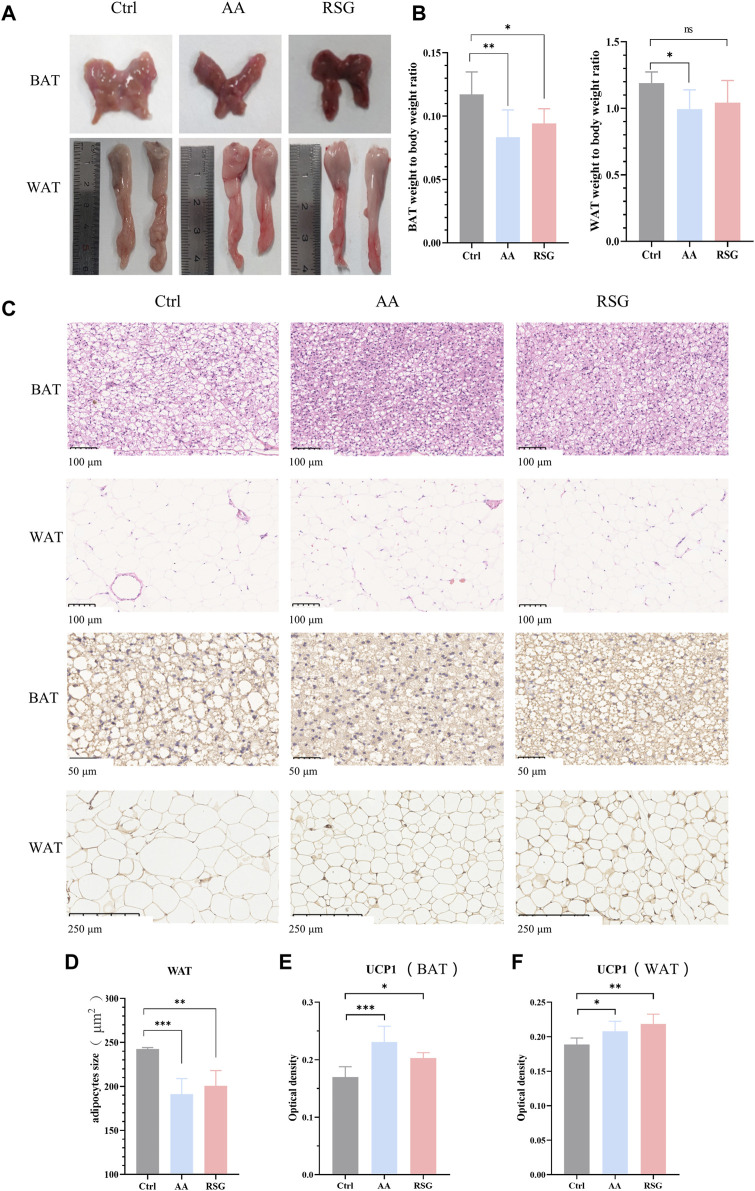
Effect of AA on BAT and WAT and the expression of UCP1. **(A)** Appearance and morphology of BAT and WAT. **(B)** Adipose tissue-to-body weight ratio. **(C)** Morphological changes in the BAT and WAT shown by HE staining (200×) and IHC staining of UCP1 in BAT (400×) and WAT (100×). **(D)** Quantifications of adipocyte sizes of WAT. **(E, F)** Quantitative analysis of UCP1 in WAT and BAT. Data are presented as mean ± SD, n = 8. *p < 0.05, **p < 0.01, and ***p < 0.001.

BAT and WAT are important regulators of energy metabolism (Cohen and Kajimura, 2021). The maintenance of sustained high metabolism relies on non-shivering thermogenesis, which is accompanied by an increase in the activity of adipose tissues (Nedergaard et al., 2001). To investigate whether the regulation of AA on energy metabolism is related to adipose tissues, we measured the weight of BAT and WAT and analyzed their morphology. Interestingly, the effect of AA on adipose tissues was particularly evident. Figure 2A shows the morphological appearance of adipose tissues. As shown in Figure 2B, the adipose weight-to-body weight ratios of the AA group were lower than that in the Ctrl group, and the BAT weight-to-body weight ratio of the RSG group was lower than that in the Ctrl group. Consistently, AA- and RSG-treated rats showed smaller adipocytes sizes, as shown by histological analysis (Figures 2C, D).

UCP1, specifically expressed in BAT, is a critical protein that generates heat (thermogenic capacity) to promote energy metabolism, and it is an indicator of WAT browning (Nedergaard et al., 2001; Machado et al., 2022). Non-shivering thermogenesis in mammals is mainly dependent on UCP1’s thermogenic capacity to boost metabolism to regulate body temperature. Therefore, we performed IHC analysis on BAT and WAT to measure the expression changes of UCP1. As expected, the expression of UCP1 was significantly upregulated in BAT and WAT of AA and RSG groups than in those of the Ctrl group (Figures 2E, F), which meant that AA enhanced the activation of BAT and browning of WAT.

### 3.3 The energy metabolism-promoting effect of AA is associated with gut microbiota

**FIGURE 3:**
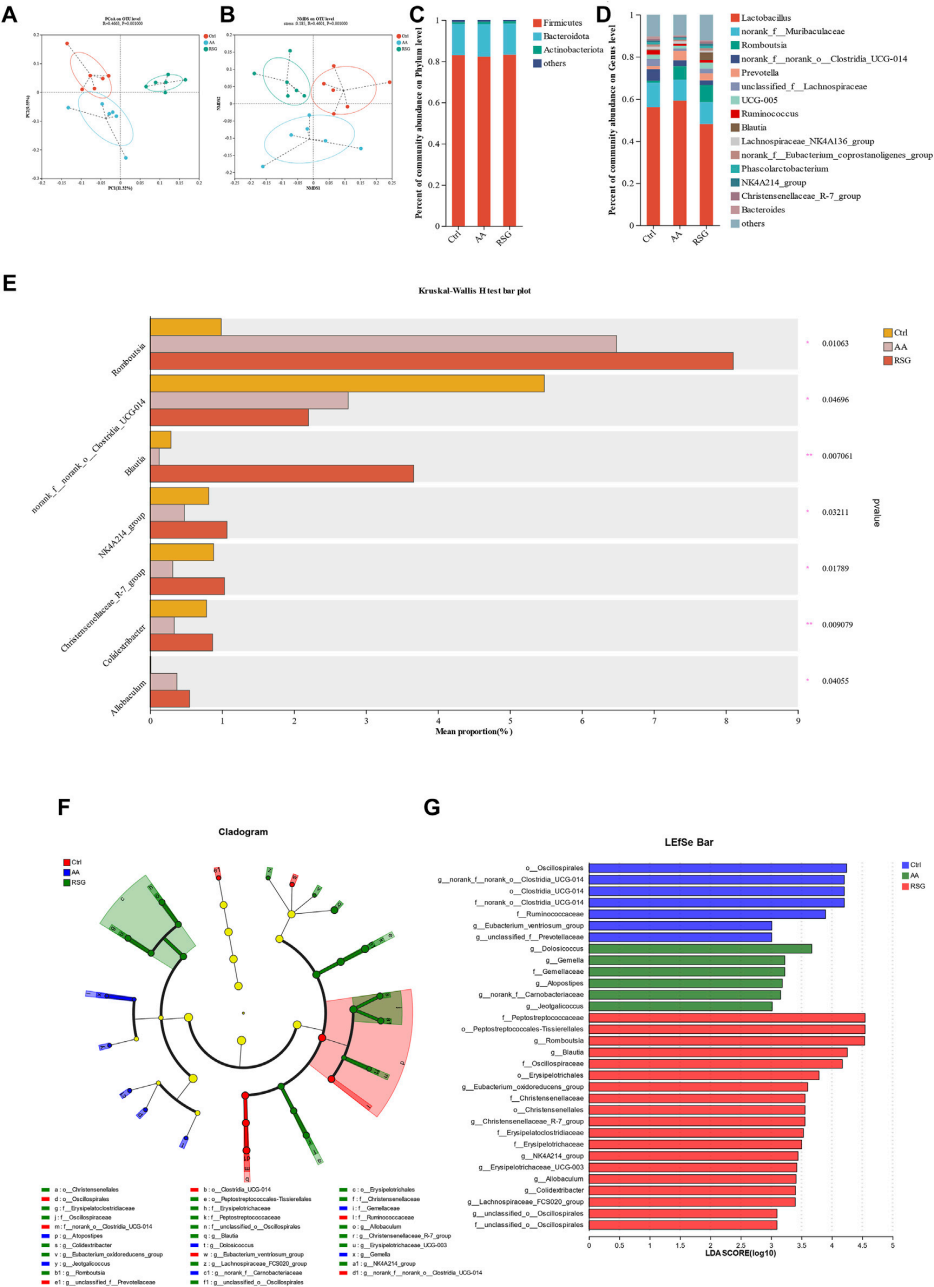
Regulatory effects of AA on gut microbiota composition. **(A)** PCoA of gut microbiota based on OTU abundance. **(B)** NMDS of gut microbiota based on OTU abundance. **(C)** Main composition of gut bacteria at the phylum level. **(D)** Main composition of gut microbiota at the genus level. **(E)** Specific differences in bacteria between different groups at the genus level. **(F)** Cladogram analysis. **(G)** LEfSe analysis of the gut microbiota between three groups. Data are presented as mean ± SD, n = 6. *p < 0.05 and **p < 0.01.

There is growing evidence that gut microbiota regulates adipose tissue and energy metabolism (Mestdagh et al., 2012; Bohan et al., 2019). The gut microbiota can also influence the whole-body metabolism by affecting the energy balance. To determine the effects of AA on gut microbiota, 16S rRNA high-throughput sequencing was performed to analyze the changes in the gut microbiota. PCoA (principal coordinates analysis) and NMDs (non-metric multidimensional scaling) based on unweighted UniFrac distances were performed to show distinct clustering of gut microbial communities for each group. A significant separation of the gut microbiomes was observed among the three groups of rats (Figures 3A, B). To identify the composition of gut microbiota and the changes in strains in each group of rats, we analyzed the gut microbiota at the phylum and genus levels (Figures 3C, D). At the phylum level, the Firmicutes and Bacteroidetes were the predominant microbiota; however, there was no significant differential tendency in the relative abundance of Firmicutes and Bacteroidetes between the three groups. At the genus level, AA increased the abundance of the *Romboutsia* and *Allobaculum*, and RSG had the same effect on these two bacteria. In addition, AA reduced the abundance of the *norank_f_norank_o_Clostridia_UGG-014*, *Blautia*, *NK4A214_group*, *Christensenellaceae_R-7_group*, and *Colidextribacter*, while RSG had the opposite effects on *Blautia*, *NK4A214_group*, *Christensenellaceae_R-7_group*, and *Colidextribacter* (Figure 3E). Then, cladogram analysis and linear discriminant analysis of the effect size (LEfSe) was used in three groups of rats to identify specific system types significantly regulated after AA and RSG treatment (LDA >3) (Figure 3G). Together, these results demonstrated that AA has a substantial effect on modulating gut microbiota.

### 3.4 The energy metabolism-promoting effect of AA is relevant to gut microbiota-derived BAs

**FIGURE 4:**
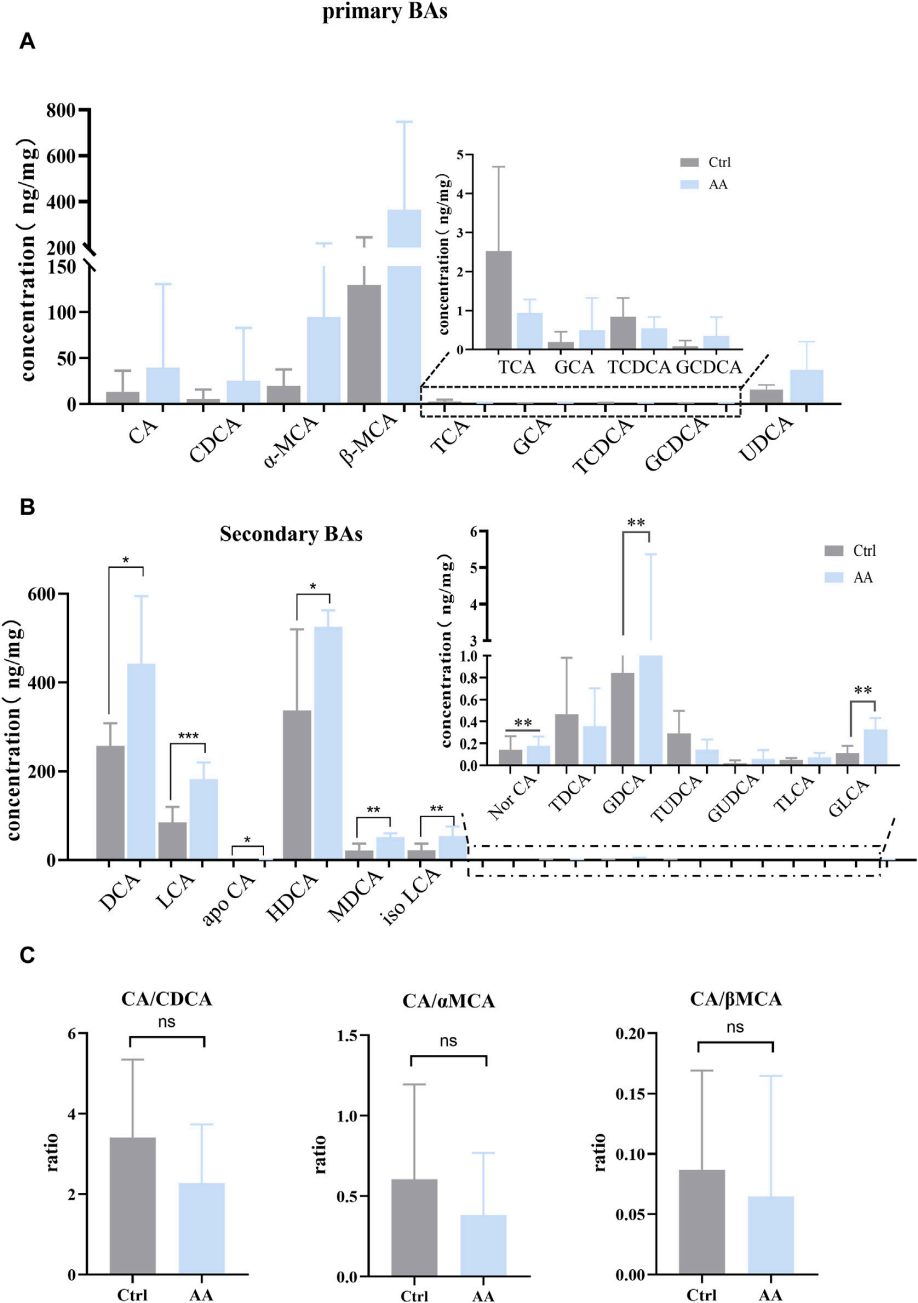
Effect of AA on fecal BA metabolism. **(A)** Effect of AA on primary BAs. **(B)** Effect of AA on secondary BAs. **(C)** Effect of AA on the BA ratio. Data are presented as mean ± SD, n = 6. *p < 0.05, **p < 0.01, and ***p < 0.001.

BAs mediated by gut microbiota are important regulators of energy metabolism (Chiang, 2013; Cai et al., 2022). To investigate whether AA affects BAs to promote energy metabolism, targeted metabolomics was used to analyze BAs in rat feces. As shown in Figure 4A, note that, except for TCA (taurocholic acid) and TCDCA (taurochenodeoxycholic acid), the effect of AA on other primary BAs showed an upward trend. Additionally, the contents of secondary unconjugated BAs ((DCA (deoxycholic acid), LCA (lithocholic acid), apoCA (apocholic acid), HDCA (hyodeoxycholic acid), MDCA (murideoxycholic acid), nor CA (norcholic acid), and isoLCA (isolithocholic acid)) and secondary conjugated BAs ((GDCA (glycodeoxycholic acid) and GLCA (glycolithocholic acid)) were significantly increased. Therefore, AA administration resulted in the conversion of primary BAs to secondary BAs, thereby massively increasing secondary BA levels.

Previous studies have revealed that the improvement of energy metabolism is accompanied by the inhibition of the classical pathway and the upregulation of the alternative pathway (Ziętak et al., 2016; Kuang et al., 2020; Han et al., 2021). Hence, we calculated CA (cholic acid)/CDCA (chenodeoxycholic acid) values in order to assess whether AA induces BA synthesis to shift from classical to alternative pathways. We also calculated the CA/*α*-MCA (alpha-muricholic acid) and CA/*β*-MCA (beta-muricholic acid) ratios since CDCA is metabolized into *α*-MCA and *β*-MCA in the liver. As shown in Figure 4C, AA administration had no significant effect on CA/CDCA, CA/*α*-MCA, and CA/*β*-MCA ratios, but there was a downward trend. This suggested that, in our experiment, AA may regulate the classical pathway rather than upregulating BA’s alternative pathway to promote energy metabolism in response to cold exposure.

**FIGURE 5:**
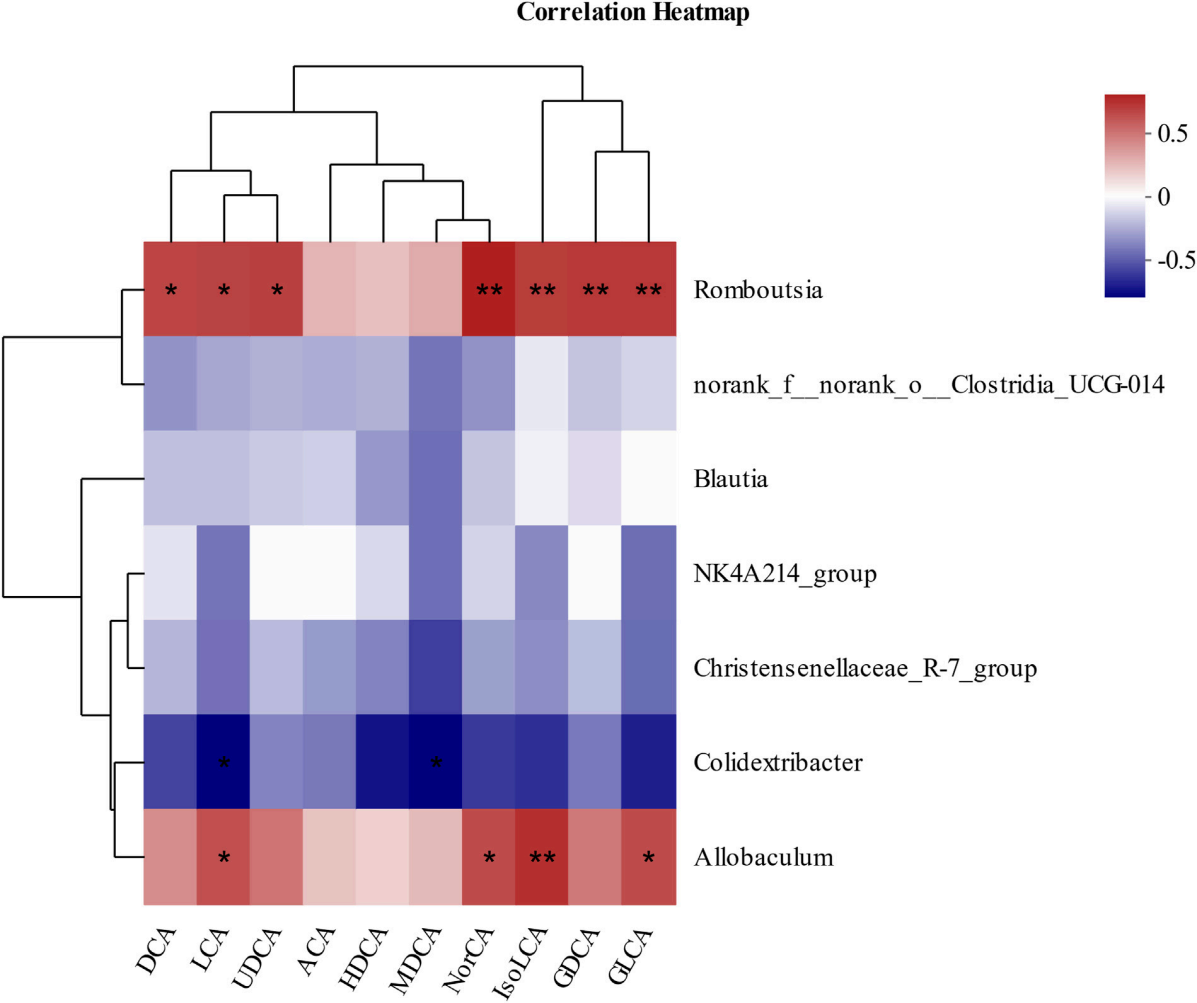
Pearson’s correlation analysis between fecal BAs and relative abundance of bacteria at the genus level. *p < 0.05, **p < 0.01, and ***p < 0.001. Red, positive correlation; blue, negative correlation.

Then, Pearson’s correlation coefficient was used to analyze the correlation between gut microbiota and BAs to visualize the potential association, and the results showed significant correlations between gut microbiota and several BAs. As shown in Figure 5, DCA, LCA, UDCA (ursodeoxycholic acid), NorCA, IsoLCA, GDCA, and GLCA were positively correlated with *Romboutsia*. In addition, LCA, NorCA, IsoLCA, and GLCA were positively correlated to *Allobaculum*. Additionally, LCA and MDCA were negatively correlated with *Colidextribacter*.

### 3.5 The energy metabolism-promoting effect of AA is associated with bile acid receptor TGR5-UCP1 signaling

**FIGURE 6:**
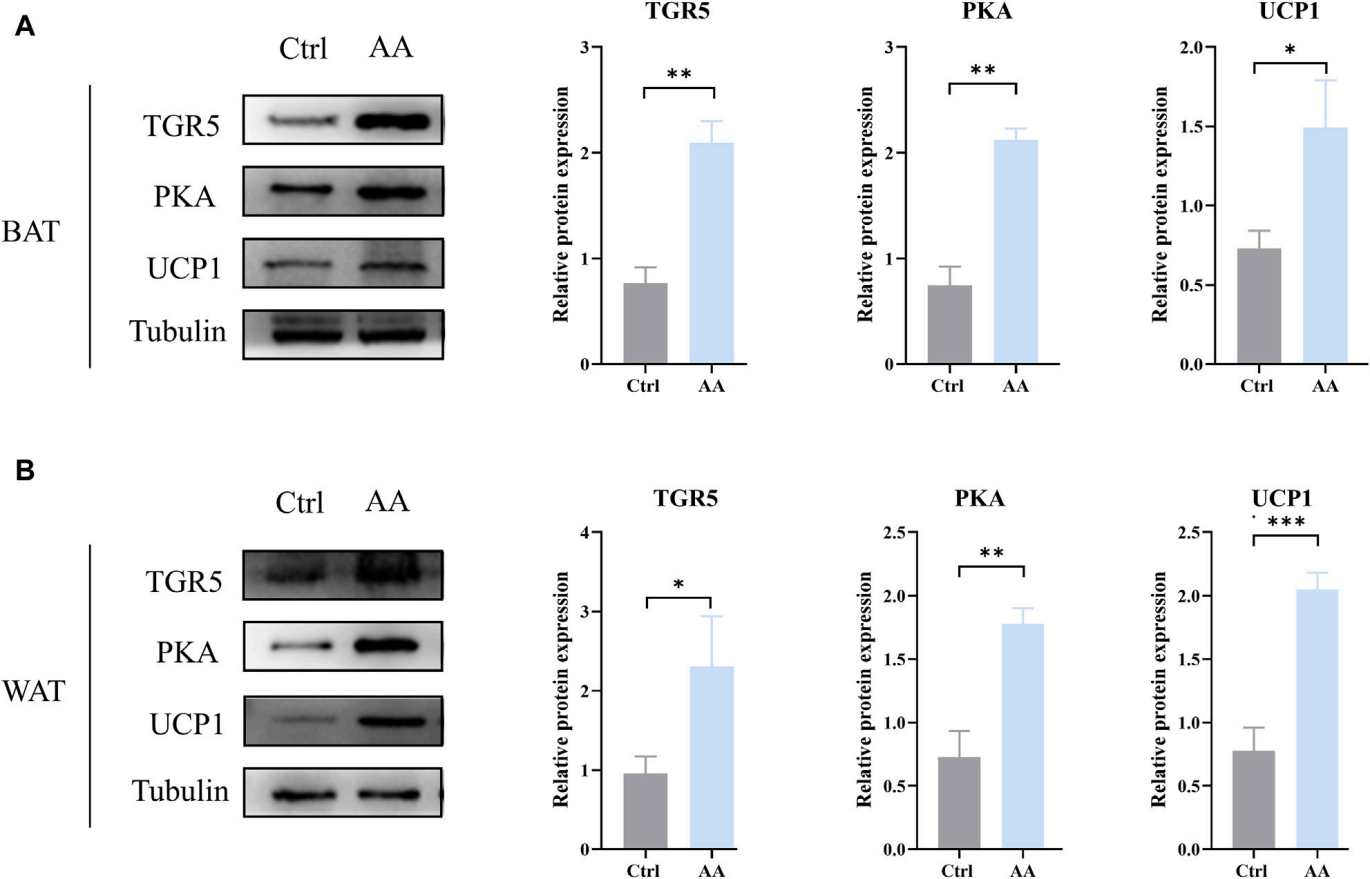
AA promoted UCP1 expression via the regulation of BAs to upregulate the cAMP/PKA signaling pathway in BAT and WAT. **(A, B)** Protein expression of TGR5, PKA, and UCP1 in BAT and WAT detected by WB. Data are presented as mean ± SD, n = 3. *p < 0.05, **p < 0.01, and ***p < 0.001.

TGR5, a specific receptor of BAs, enhances UCP1 thermogenesis in BAT and WAT via stimulation of cAMP/PKA signaling pathways, thereby promoting energy metabolism (Thomas et al., 2008; Cai et al., 2022; Perino and Schoonjans, 2022). To detect whether AA promotes energy metabolism through the thermogenic signaling pathway chain TGR5-cAMP/PKA-UCP1, we examined their expression in adipose tissue. As depicted in Figure 6, upregulation of TGR5, PKA, and UCP1 can be observed in BAT and WAT, which indicated the enhanced thermogenesis-promoting effect of BAT and browning of WAT. Taken together, the promotion of energy metabolism by AA is connected to the TGR5-UCP1 signaling pathway in adipose tissue.

### 3.6 The absence of gut microbiota weakened the energy metabolism-promoting effect of AA

**FIGURE 7:**
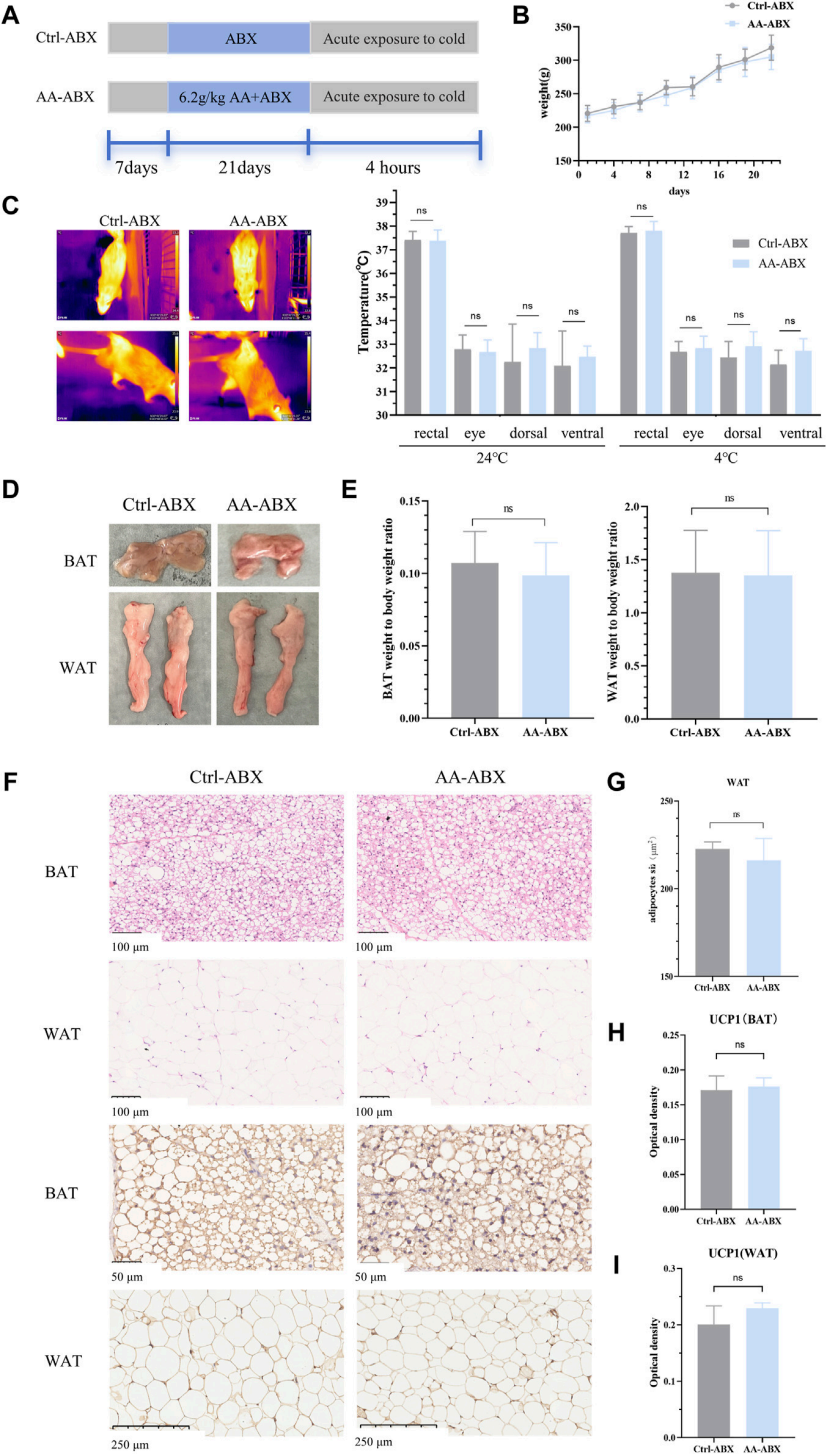
Effect of AA on the metabolic phenotype in pseudo-germ-free rats. **(A)** Experimental design of the pseudo-germ-free experiment. **(B)** Body weight of each group of rats. **(C)** Representative thermal images of rats and body temperature of rats before and after cold exposure. **(D)** Adipose tissue morphology of BAT and WAT. **(E)** Adipose tissue-to-body weight ratio. **(F)** Morphological changes in the BAT and WAT shown by HE staining (200×) and IHC staining of UCP1 in the BAT (400×) and WAT (100×). **(G)** Quantifications of adipocyte sizes of WAT. **(H, I)** Expression of UCP1 in the BAT and WAT. Data are presented as mean ± SD, n = 8. *p < 0.05, **p < 0.01, and ***p < 0.001.

In order to determine whether the gut microbiota is a pivotal part of AA in promoting energy metabolism, a pseudo-germ-free rat model treated with antibiotics was used (Figure 7A). As shown in Figure 7B, the body weight of rats had no significant differences between the Ctrl-ABX group and AA-ABX group. In addition, the rectal temperature and temperature of different body surface sites were also measured before and after cold exposure. The results showed that after the elimination of gut microbiota, the core body temperature and surface temperature of the AA-ABX and Ctrl-ABX group rats were similar at room temperature. In addition, consistent with changes in body temperature at room temperature, the core body temperature and surface temperature of the AA-ABX and Ctrl-ABX group rats showed no difference upon cold exposure. Furthermore, there was no significant variation in the ratio of adipose tissue to body weight between the two groups (Figure 7E). Histological results showed that the adipose tissue diameter of the AA-ABX group was not significantly reduced compared with that of the Ctrl-ABX group (Figures 7F, G). Aligned with these results, the ability of AA to enhance UCP1 expression in BAT and WAT was weakened in microbiota-eliminated rats (Figures 7H, I). These results revealed that the pro-energy metabolic benefits of AA were attenuated in the absence of gut microbiota, further suggesting that the gut microbiota is essential for the function of AA.

### 3.7 Effects of microbial transplantation from AA on body weight and body temperature

**FIGURE 8:**
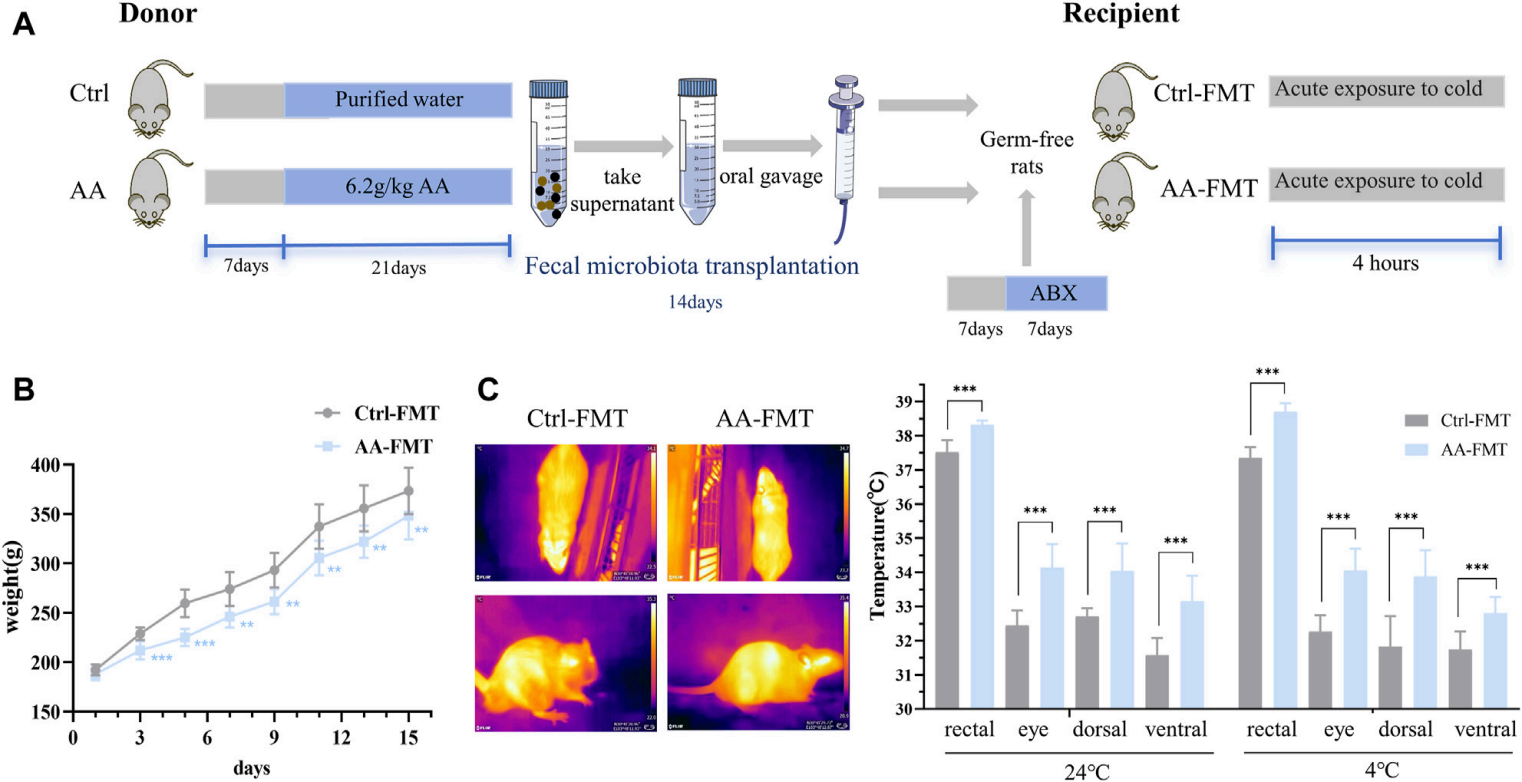
Effects of FMT from AA on the body weight and body temperature in rats. **(A)** Experiment design of the FMT experiment. **(B)** Changes in the body weight of rats. **(C)** Representative thermal images of rats and body temperature of rats before and after cold exposure. Data are presented as mean ± SD, n = 8. *p < 0.05, **p < 0.01, and ***p < 0.001.

After identifying the gut microbiota as the key to AA thermogenesis, we conducted FMT experiments to assess whether the rat phenotype would be recapitulated after FMT (Figure 8A). Specifically, antibiotics were given to create a pseudo-germ-free environment, and the gut microbiota of the Ctrl and AA groups rats were transferred to two separate groups of rats. After 2 weeks of FMT, there were significant differences in phenotypes between the Ctrl-FMT and AA-FMT groups. As shown in Figure 8B, the body weight of the rats in the AA-FMT group decreased after receiving the microbiota of the AA group. Additionally, FMT from AA mimicked the effect of AA on body temperature in rats. As shown in Figure 8C, increases in core body temperature and other site temperature were observed in the AA-FMT group of rats at room temperature. After cold exposure, the temperature of rats in the AA-FMT group was still higher than that in the Ctrl-FMT group.

### 3.8 Microbial transplantation from AA on the activity of BAT and browning of WAT

**FIGURE 9:**
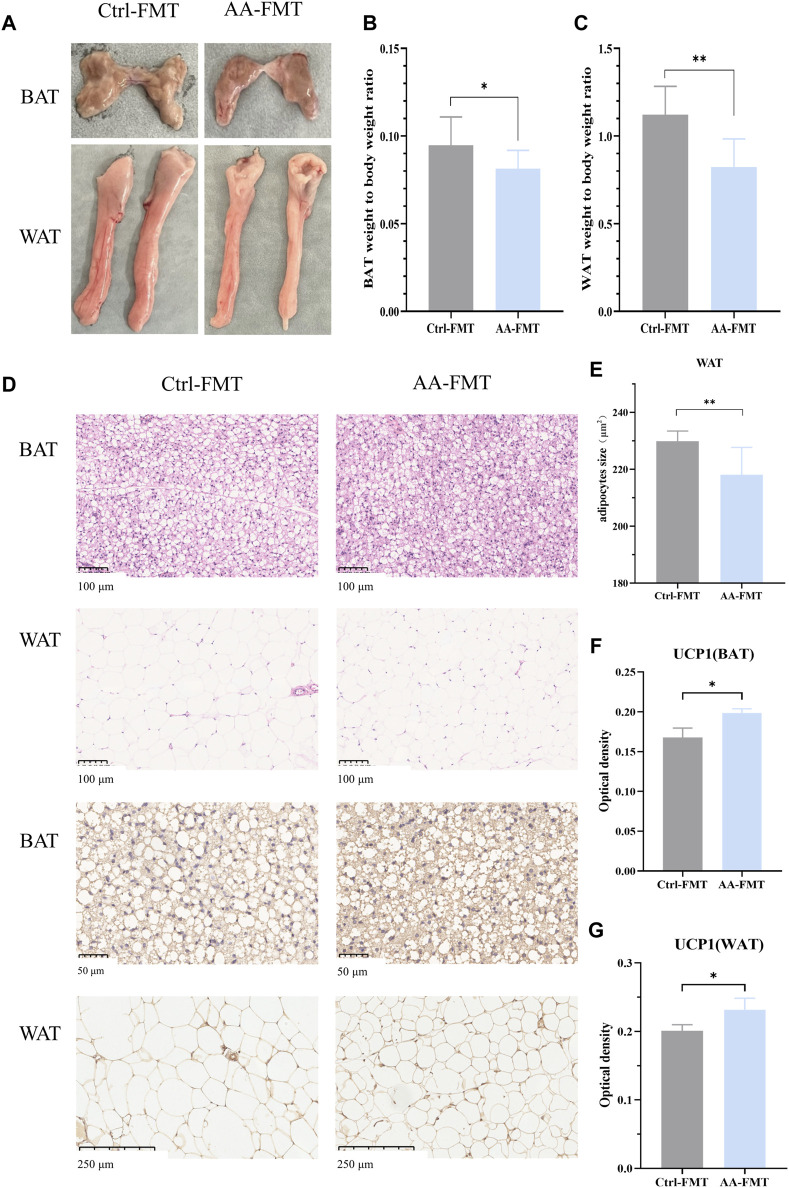
Effects of FMT from AA on adipose tissues in rats. **(A)** Adipose tissue morphology of BAT and WAT. **(B, C)** Adipose tissue-to-body weight ratio. **(D)** Morphological changes in the BAT and WAT shown by HE staining (200×) and IHC staining of UCP1 in the BAT (400×) and WAT (100×). **(E)** Quantifications of adipocyte sizes of WAT. **(F, G)** Expression of UCP1 in the BAT and WAT. Data are presented as mean ± SD, n = 8. *p < 0.05 and ***p < 0.001.

After 14 days of FMT, we found that the effects of AA on adipose tissue can be recapitulated after FMT. The adipose tissues of the two groups of rats after FMT are shown in Figure 9A. As shown in Figures 9B, C, the ratio of adipose tissue to body weight is lower than that of Ctrl-FMT. Histological analysis showed that adipocytes of the AA-FMT group appeared smaller relative to those of the Ctrl-FMT group (Figures 9D, E). IHC analysis showed that AA still appeared to have a positive regulatory effect on UCP1 in adipose tissue after FMT (Figure 9D). As shown in Figures 9F, G, the expression of UCP1 in the AA-FMT group was significantly upregulated after cold exposure compared with the Ctrl-FMT group.

### 3.9 Microbial transplantation from AA on the gut microbiota

**FIGURE 10:**
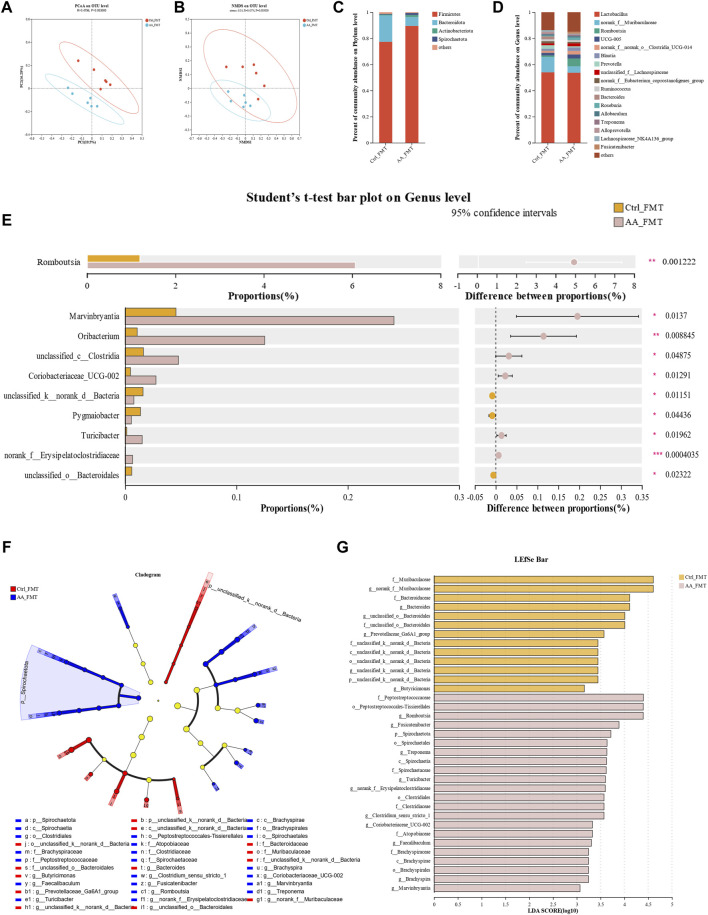
Effect of FMT of AA on gut microbiota in rats. **(A)** PCoA of gut microbiota based on OTU abundance. **(B)** NMDS of gut microbiota based on OTU abundance. **(C)** Main composition of intestinal bacteria at the phylum level. **(D)** Main composition of gut microbiota at the genus level. **(E)** Specific differences in bacteria between different groups at the genus level. **(F)** Cladogram analysis. **(G)** LEfSe analysis of the gut microbiota between three groups. Data are presented as mean ± SD, n = 6. *p < 0.05, **p < 0.01, and ***p < 0.001.

Next, we studied the structure of the gut microbiota of rats transplanted with fecal microbiota to confirm the successful transfer of the gut microbiota. PCoA and NMDS based on unweighted UniFrac distances results showed that a clear separation in the intestinal microbial communities was observed between Ctrl-FMT and AA-FMT groups (Figures 10A, B). Figures 10C, D showed the microbiota composition of the Ctrl-FMT and AA-FMT groups at the phylum and genus levels, respectively. At the phylum level, the Firmicutes and Bacteroidetes accounted for the largest proportion, which was in line with previous phenotypic findings. Further analysis showed that there were differences in microbial composition between the two groups at the genus level. As shown in Figure 10E, the rats in the AA-FMT group had significantly increased relative abundance of *Romboutsia*, *Marvinbryantia*, *Oribacterium*, *unclassified_c_Clostridia*, *Coriobacteriaceae_UCG_002*, *Turicibacter*, and *norank_f_Erysipclatoclostridiaccac* and lowered the relative abundance of *unclassified_k_norank_d_Bacteria*, *Pygmaiobacter*, and *unclassified_o_Bacteroidales* when compared with the Ctrl-FMT group. In addition, cladogram analysis and LEfSe were used in two groups of rats to identify specific system types significantly regulated after FMT (LDA >3) (Figures 3F, G). Together, these results demonstrated that FMT from AA had a substantial effect on regulating gut microbiota.

### 3.10 Effects of microbial transplantation from AA on the gut microbiota-derived BAs

**FIGURE 11:**
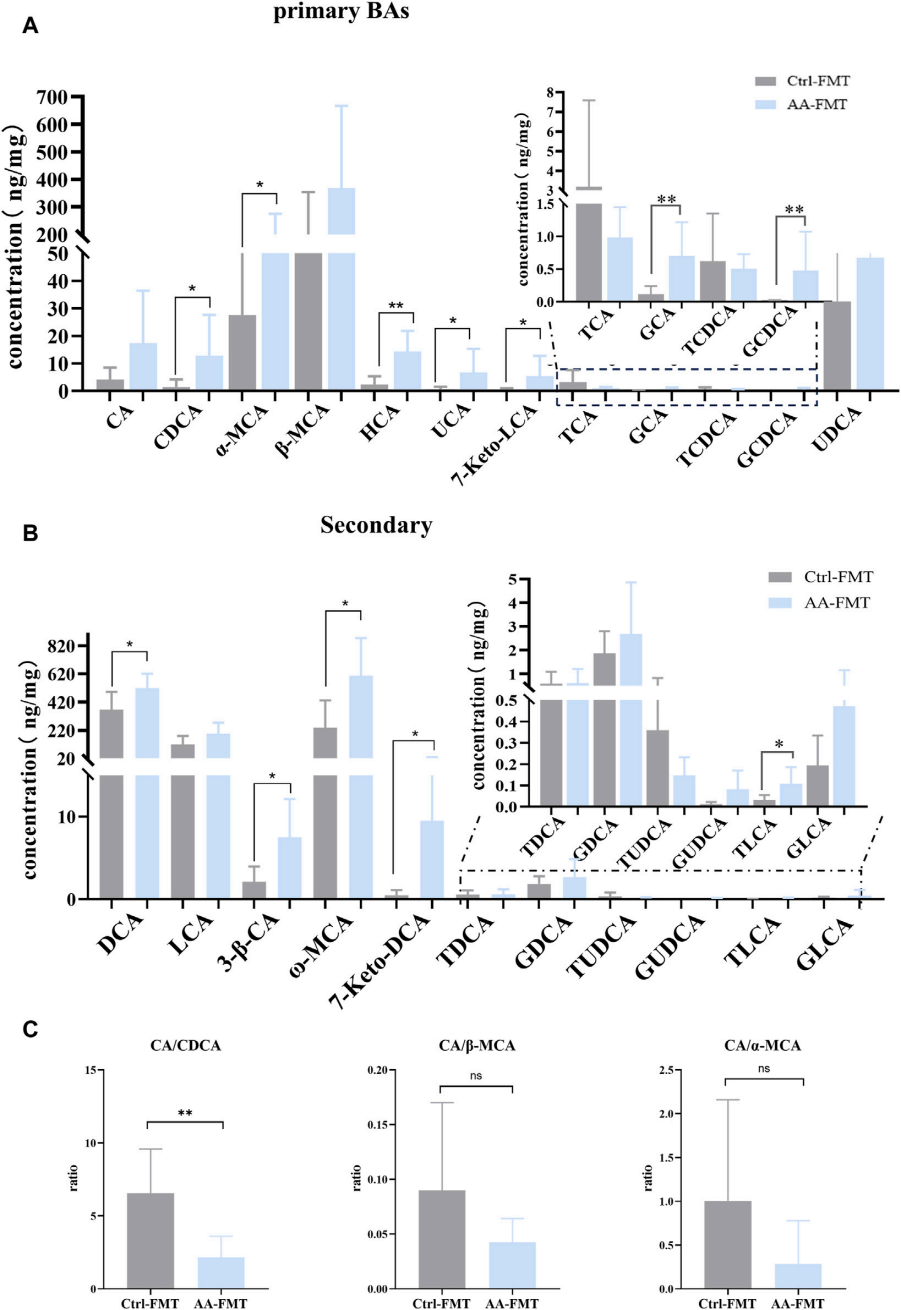
Effect of FMT of AA on fecal BA metabolism. **(A)** Effect of FMT of AA on primary BAs. **(B)** Effect of FMT of AA on secondary BAs. **(C)** Effect of FMT of AA on the BA ratio. Data are presented as mean ± SD, n = 6. *p < 0.05 and **p < 0.01.

**FIGURE 12:**
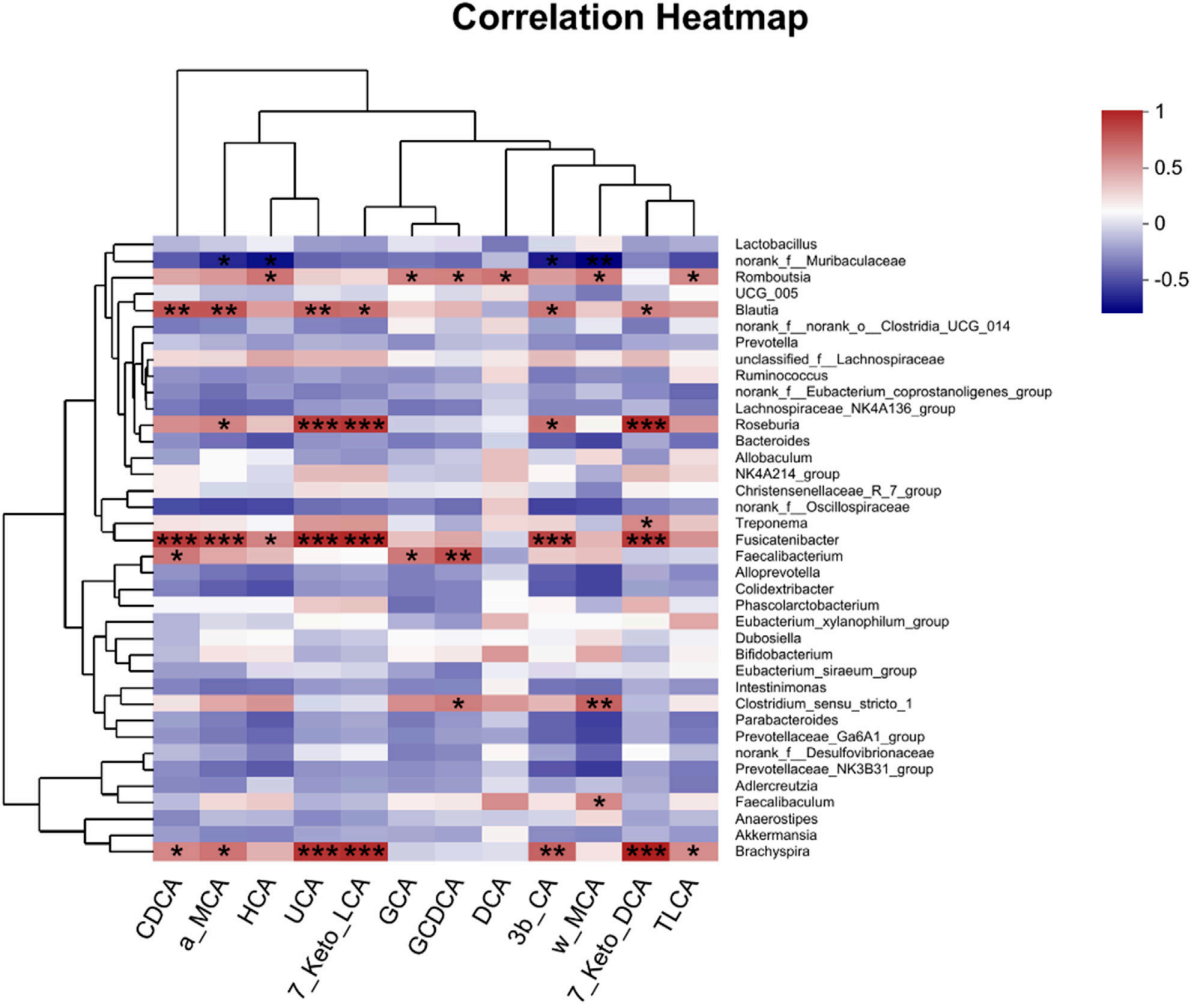
Pearson’s correlation analysis between fecal BAs and relative abundance of bacteria at the genus level. *p < 0.05, **p < 0.01, and ***p < 0.001. Red, positive correlation; blue, negative correlation.

To investigate whether AA-induced microbial transfer affects BA conversion, we again examined fecal BAs from Ctrl-FMT and AA-FMT rats. As shown in Figure 11A, compared with Ctrl-FMT rats, increases of primary unconjugated BAs CDCA, *α*-MCA, HCA (hyocholic acid), UCA (ursocholic acid), 7 keto-LCA, primary conjugated BA GCA (glycocholic acid), and GCDCA (glycochenodeoxycholic acid) were observed in the AA-FMT group. Furthermore, significant increases in secondary unconjugated BAs DCA, 3*β*-CA (3*β*-cholic acid), ω-MCA, 7 keto-DCA, and secondary conjugated BAs TLCA (taurolithocholic acid) were also observed (Figure 11B). Interestingly, the increase in DCA was consistent with the trend in the donor group. In line with the above, we also calculated their ratio of CA/CDCA, CA/*α*-MCA, and CA/*β*-MCA. As shown in Figure 11C, a significant reduction in the CA/CDCA ratio was observed in the AA-FMT group. In addition, downregulated CA/*α*-MCA and CA/*β*-MCA ratios were observed, even though the values were not significantly different. This suggests that AA after FMT also tended to regulate the classical pathway to promote energy metabolism. In addition, Pearson’s correlation coefficient showed that HCA, GCA, GCDCA, DCA, ω-MCA, and TLCA had a positive correlation with *Romboutsia*, and CDCA, *α*-MCA, UCA, 7Keto-LCA, 3*β*-CA, and 7Keto-DCA had a positive correlation with *Blautia*, *Roseburia*, *Fusicatenibacter*, and *Brachyspira* (Figure 12); the results showed that there was a significant correlation between gut microbiota and BAs.

### 3.11 Effects of microbial transplantation from AA on the BA receptor TGR5-UCP1 signaling pathway

**FIGURE 13:**
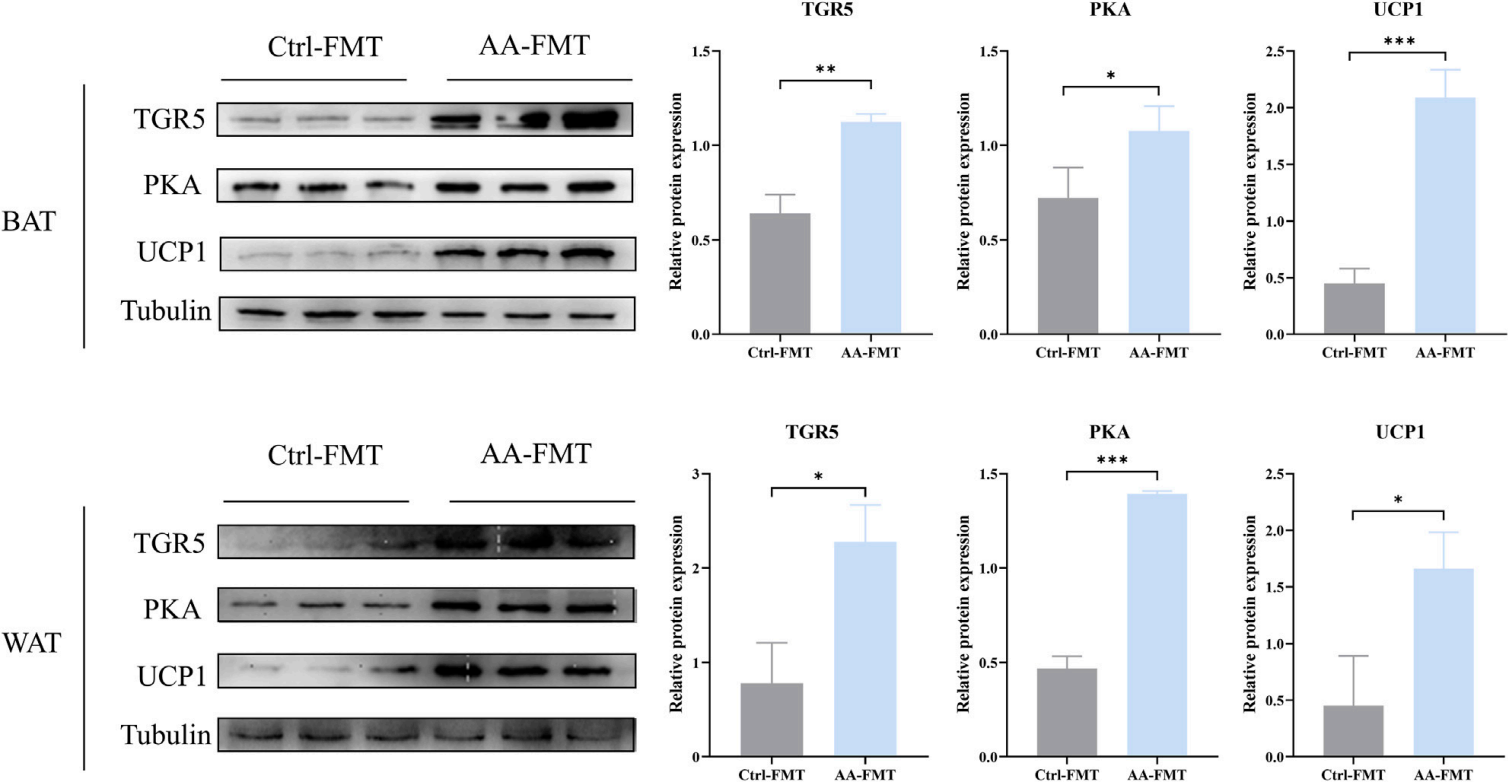
FMT of AA can also promote UCP1 expression via the regulation of BAs to upregulate the cAMP/PKA signaling pathway in BAT and WAT. Data are presented as mean ± SD, n = 3. *p < 0.05, **p < 0.01, and ***p < 0.001.

To determine whether AA can regulate the TGR5-cAMP/PKA-UCP1 signaling pathway after FMT, we also detected their expressions in BAT and WAT via WB. As shown in Figure 13, the AA-FMT group significantly upregulated the expressions of TGR5, PKA, and UCP1 in BAT and WAT.

## 4 Discussion

Cold and hot properties are one of the guiding principles of Chinese medicine in the treatment of diseases. As a classical TCM with a hot nature, aconite can reinforce warming and dispel cold to enhance the body temperature (Yu et al., 2011; Zhou et al., 2015). Our previous study has confirmed that aconite can increase the body temperature of rats when indirectly exposed to severe cold (swimming at low temperature) (Liu et al., 2021). Here, we placed rats in a 4°C freezer for cold exposure to assess whether AA can promote energy metabolism. The results showed that AA administration could increase the body temperature of rats (rectal, eye, dorsal, and ventral) at both room temperature and cold exposure. Furthermore, our results indicated that the regulation of gut microbiota by AA led to changes in BA metabolism, which led to the activation of TGR5-UCP1 signaling in BAT and WAT.

Gut microbiota, as a factor in regulating fat storage and utilization, plays an important regulatory role in the process of energy metabolism (Heiss and Olofsson, 2018). Pseudo-germ-free models treated with ABX and germ-free models have been widely used to explore the relationship between gut microbiota and energy. A previous experiment showed that different ABX mixtures resulted in impaired thermogenic function of BAT and browning function of WAT. Interestingly, the use of germ-free models further revealed similar findings (Li et al., 2019). In addition, an experiment on resveratrol in combination with ABX treatment showed that no WAT browning and enhanced BAT activity were detected when the gut microbiota was eliminated (Hui et al., 2020). In addition, additional naringenin-induced FMT experiments confirmed that gut microbiota promoted the browning of beige adipose to produce heat upon cold stimulation (Zhang et al., 2022). Hence, both antibiotic treatment and FMT further revealed the important role of the gut microbiota. Due to the low bioavailability of aconite and its potential in being a substrate for gut microbiota, we hypothesized that aconite might induce the function of BAT and WAT via the regulation of gut microbiota. In line with the expectation, AA induced thermogenesis in BAT and browning in WAT to promote energy metabolism, which was accompanied with variation in gut microbiota, including the enhanced abundance of the *Romboutsia* and *Allobaculum* and the reduced abundance of the *norank_f_norank_o_Clostridia_UGG-014*, *Blautia*, *NK4A214_group*, *Christensenellaceae_R-7_group*, and *Colidextribacter*. However, the advantageous effects of AA on energy metabolism were not discernible after combining ABX with AA, such as less weight, increased body temperature, morphological changes of BAT and WAT, and high expression of UCP1. After performing FMT, similar outcomes regarding the positive impact of AA on the donor group were observed once more. The AA-FMT group, the receivers of AA microbiota, showed a reduction in body weight, increase in temperature, enhancement in BAT and WAT activities, and the higher expression of UCP1. Meanwhile, it was accompanied by changes in the gut microbiota, including increased relative abundance of *Romboutsia*, *Marvinbryantia*, *Oribacterium*, *unclassified_c_Clostridia*, *Coriobacteriaceae_UCG_002*, *Turicibacter*, and *norank_f_Erysipclatoclostridiaccac* and lowered the relative abundance of *unclassified_k_norank_d_Bacteria*, *Pygmaiobacter*, and *unclassified_o_Bacteroidales*. Although our two experiments did not replicate exactly the same changes in the gut microbiota, the role of the gut microbiota cannot be ignored.

The liver–microbiota metabolic axis is the focus of research, and the gut microbiota influences the microbiota metabolites to mediate the regulation of energy metabolism. BAs, as biologically active signaling molecules, control the body’s metabolism by activating corresponding receptors. Previous research studies have found that alterations in gut microbiota increased the production of BAs during cold exposure, which contributed to increasing the thermogenic effect of BAT and WAT (Ziętak et al., 2016; Worthmann et al., 2017). Similarly to those of BAs under cold exposure, we found that AA significantly increased the content of secondary unconjugated BAs (DCA, LCA, apoCA, HDCA, MDCA, Nor CA, and isoLCA) and secondary conjugated BAs (GDCA and GLCA). In addition, the AA-FMT group also took on changes in BA levels, mainly including increased levels of primary unconjugated BAs such as CDCA, *α*-MCA, HCA, UCA, and 7Keto-LCA; primary conjugated BAs such as GCA and GCDCA; secondary unconjugated BAs such as DCA, 3*β*-CA, *ω*-MCA, and 7Keto-DCA; and secondary conjugated BAs such as TLCA. In addition, accumulated studies have shown that inhibition of the classical pathway and upregulation of the alternative pathway are important characteristics of energy metabolism improved by BAs (Ziętak et al., 2016; Sun et al., 2019; Kuang et al., 2020), which is positively correlated with the promotion in metabolic diseases. To explain the specific effects of AA’s promotion of energy metabolism on the classical and alternative pathways, we assessed the proportion of secondary BAs corresponding to these two pathways. However, contrary to previous findings, our results did not present the inhibition of classical pathways or the promotion of alternative pathways, but they were more inclined to present the upregulation of classical pathways. One of the reasons for this may be that our experiment was based on a normal animal model, whereas previous studies were on an obese one. It should be noted that the role of alternative approaches cannot be ignored. Secondary BAs (LCA and DCA) are natural agonists of TGR5. TGR5 has a significant effect on adipose tissue and is important for how the gut microbiota and host metabolism work together (Chen et al., 2011; van Nierop et al., 2017). The combination of BAs and TGR5 triggers the cAMP-PKA signaling pathway, which is conducive to promoting UCP1 expression (Thomas et al., 2008; Molinaro et al., 2018; Wei et al., 2020; Perino and Schoonjans, 2022). However, activation of the cAMP/PKA signaling pathway was eliminated in BAT and WAT after the knockout of TGR5 (Han et al., 2021). In order to further analyze the effects of AA on TRG5, PKA, and UCP1, we detected their expressions from the protein level by WB. The results showed that in the AA and AA-FMT groups, the protein content of TGR5, PKA, and UCP1 increased. This revealed that the energy metabolism-promoting effect of aconite is associated with gut microbiota and bile acid receptor TGR5-UCP1 signaling.

Interestingly, there was an increase in *Romboutsia* and DCA in both oral AA and AA-FMT. Pearson’s correlation analysis showed that *Romboutsia* was positively correlated with DCA. *Romboutsia* has been previously reported to be related to energy metabolism and BAs (Liu et al., 2018; Zhao et al., 2024). Increased levels of DCA have been shown to activate fat TGR5 signaling to further promote thermogenesis (Somm et al., 2017; Wu et al., 2021). Surprisingly, AA and AA-remodeled FMT administration both resulted in reduced TCA and TCDCA and increased DCA, UDCA, and LCA. TCA and DCA are the primary taurine conjugate and secondary unconjugate form of CA in the classical pathway, respectively. TCDCA is the primary taurine conjugate of CDCA in the alternative pathway, and LCA is the secondary unconjugated form of CDCA. The paradoxical result may be due to changes in the gut microbiota.

In summary, our study confirmed that after AA intervention, the modified gut microbiota influenced the conversion of primary to secondary metabolism of BAs, which may lead to changes in the pathways of BA synthesis in the liver and increased energy expenditure in adipose tissue. Consequently, the regulation of the gut microbiome–BA axis via AA could serve as a potential regulator for reducing obesity.

## 5 Conclusion

**FIGURE 14:**
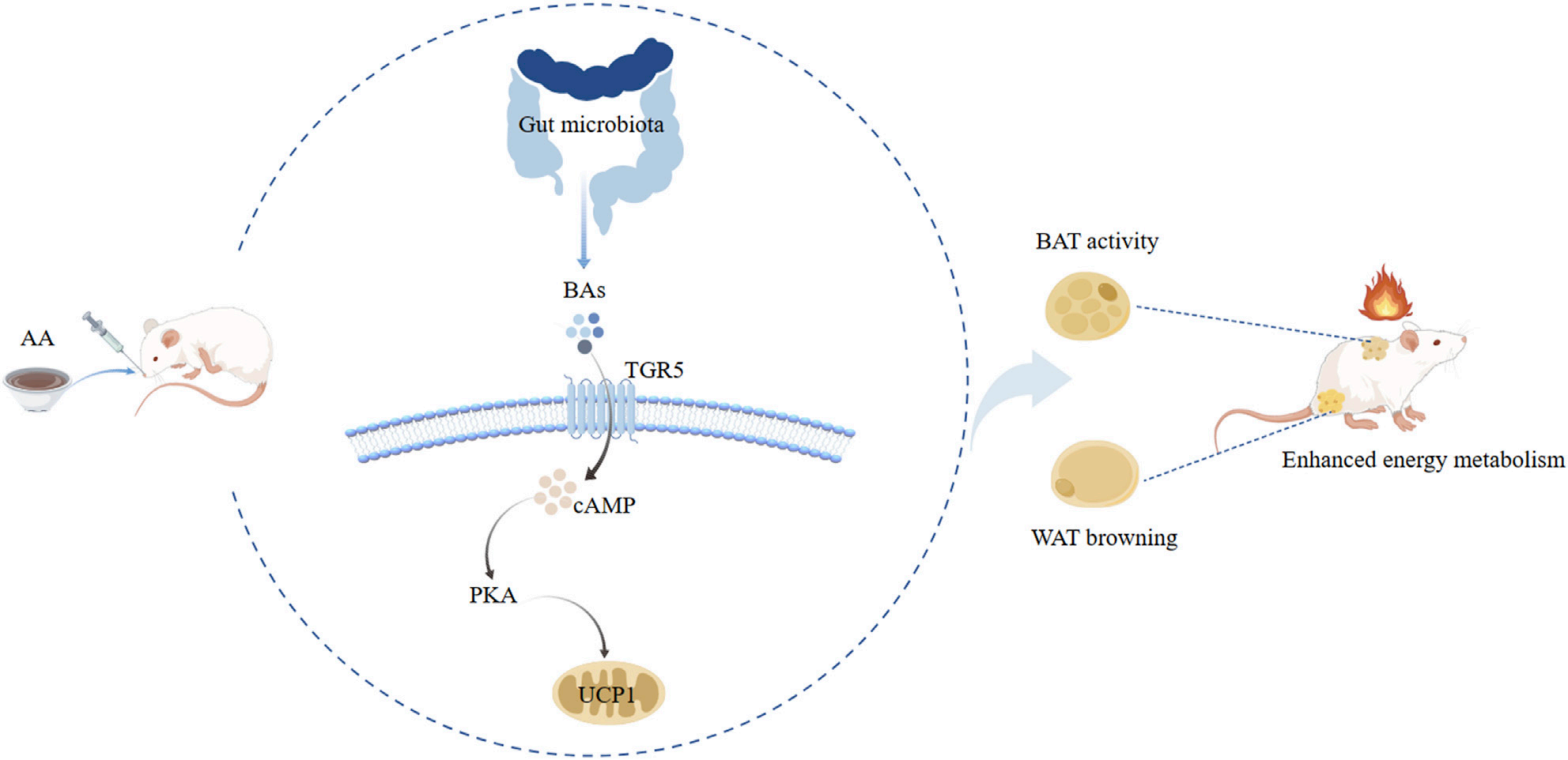
Potential mechanism analysis of AA promoting energy metabolism along the gut microbiota–BA–adipose tissue axis.

We discovered a mechanism by which aconite promoted energy metabolism. Gut microbiota and BAs mediated the UCP1 thermogenesis of AA to enhance the activity of BAT and promote the browning of WAT. Subsequent analysis showed that FMT from AA can also regulate gut microbiota and BA metabolism. Taken together, our study revealed that the energy metabolism-promoting effect of aconite is associated with gut microbiota and bile acid receptor TGR5-UCP1 signaling (Figure 14).

## Figures and Tables

**Table:** 

**WAT**	white adipose tissue
**BAT**	brown adipose tissue
**BAs**	bile acids
**TGR5**	Takeda G-protein-coupled receptor 5
**ABX**	antibiotics cocktail
**FMT**	fecal microbiota transplantation
**cAMP**	cyclic adenosine monophosphate
**PKA**	protein kinase A
**TCM**	traditional Chinese medicine
**AA**	aconite aqueous extract
**RSG**	rosiglitazone
**UCP1**	uncoupling protein 1
**PCoA**	principal coordinates analysis
**NMDs**	non-metric multidimensional scaling
**CA**	cholic acid
**CDCA**	chenodeoxycholic acid
***α*-MCA**	alpha-muricholic acid
***β*-MCA**	beta-muricholic acid
**TCA**	taurocholic acid
**GCA**	glycocholic acid
**TCDCA**	taurochenodeoxycholic acid
**GCDCA**	glycochenodeoxycholic acid
**DCA**	deoxycholic acid
**UDCA**	ursodeoxycholic acid
**LCA**	lithocholic acid
**apoCA**	apocholic acid
**HDCA**	hyodeoxycholic acid
**MDCA**	murideoxycholic acid
**isoLCA**	isolithocholic acid
**Nor CA**	norcholic acid
**TDCA**	taurodeoxycholate acid
**GDCA**	glycodeoxycholic acid
**TUDCA**	tauroursodeoxycholic acid
**GUDCA**	glycoursodeoxycholic acid
**TLCA**	taurolithocholic acid
**GLCA**	glycolithocholic acid
**HCA**	hyocholic acid
**UCA**	ursocholic acid
**7-keto-LCA**	7-ketolithocholic acid
**3*β*-CA**	3*β*-cholic acid
**7-keto-DCA**	7-ketodeoxycholic acid

## Data Availability

The datasets presented in this study can be found in online repositories. The names of the repository/repositories and accession number(s) can be found in the article/Supplementary Material.
